# Gluten-Free Products: From Dietary Necessity to Premium Price Extraction Tool

**DOI:** 10.3390/nu11091997

**Published:** 2019-08-23

**Authors:** Maria Teresa Gorgitano, Valeria Sodano

**Affiliations:** 1 Department of Agriculture, University of Naples Federico II, 80055 Portici, Italy; 2 Department of Political Science, University of Naples Federico II, 80138 Napoli, Italy

**Keywords:** gluten-free diet, celiac disease, product differentiation, hedonic price model, gluten-free pasta, Italian food retailing, healthy claims

## Abstract

Every year, the Italian National Health Service (NHS) provides about 200,000 celiac people (based on 2017 data) living in Italy with financial support of about 250 million euro to cover the cost of their specific dietary constrains. The existence of gluten-free products of high quality and affordable price is very important for the quality of life of celiac people and the sustainability of public support. Over the last decade, the market for gluten-free products has experienced a dramatic surge, with an increasing shelf space dedicated to these products in supermarkets, and a large variety of products both in terms of kind of agricultural inputs and processing and packaging methods. This study aimed at assessing the offer of gluten-free (GF) pasta in Italian supermarkets, with respect to its ability to meet the needs of celiac people in terms of variety, prices and safety. A hedonic price analysis was performed. Results indicated that GF pasta is sold only in 44% of the 212 stores of the sample, with a price equal to more than twice that of conventional pasta. A premium price was found for the following attributes: small packages, brands specialized in GF products, content in fiber and the presence of quinoa as ingredient.

## 1. Introduction

For people affected by celiac disease (CD) the avoidance of gluten is extremely important for reducing disease symptoms and improving their life expectancy. In order to meet the needs of celiac people, gluten-free food products should be easily accessible. While in the past, foods traditionally made from wheat such as bread, pasta and all the kinds of bakery products were forbidden to celiac people, over the last two decades, more and more gluten-free (GF) substitutes of these products have entered the market. At first, GF products were sold in specialty stores for diet foods. Subsequently they entered the assortment of common supermarkets, where they now are on the shelf next to their own equivalents containing gluten, or in separate display areas that bring together all types of GF products. The sale of gluten-free products is regulated through specific provisions on their definition and labelling. The European Union (EU) sets rules on GF products through Regulation (EU) No. 609/2013 and Regulation (EU) No. 1169/2011. Regulation (EU) No. 1169/2011 sets out rules on information to be provided for all food, on the presence of ingredients, such as gluten-containing ingredients, with a scientifically proven allergenic or intolerance effect. The implementing Regulation (EU) No. 828/2014 asserts that the statement ‘gluten-free’ may only be made when the food as sold to the final consumer contains no more than 20 mg/kg of gluten; while the statement ‘very low gluten’ applies to food containing no more than 100 mg/kg of gluten.

In Italy, people with celiac disease receive a monthly expense contribution for the purchase of the gluten-free products included in the National Register (NR)’s list of foods suitable for celiac people drawn up by the Italian Ministry of Health (IMH). Products included in the NR may also be voluntarily marked with a symbol provided by the IMH. In Italy, there is also a private organization, the Italian Association for Celiac Disease (AIC), that provides celiac people with help and advice for following a correct diet. The AIC also monitors the reliability of GF products and publishes a list (called ‘prontuario’) of monitored GF products; products included in the AIC prontuario can voluntarily place a symbol on the label showing a crossed grain. Both the symbols provided by the IMH and AIC have the aim of informing and guaranteeing celiac people of the accuracy and safety of their food purchases. The National Health Service (NHS) implements support policies for people with celiac disease everywhere, aimed at achieving the following objectives: ensuring easy accessibility to the purchase of GF products; guaranteeing their safety, i.e., the absence of gluten contamination, which may occur also in naturally GF products where gluten is often used as an additive; monitor the adequacy of the diet followed by celiac people, also in terms of correct nutritional intake; contribute to the additional expense that people with celiac disease have to bear for their diet, due to the high prices of many GF products. In Italy celiac people account for 0.34% (of which: female, 71%; male, 29%) of the whole population. In support of their gluten-free diet, in 2017, the NHS spent about 250 million euro on gluten-free products, with an annual national average of around €1200.00 per capita [1].

Over the recent years, many studies have been carried out attempting to evaluate how much celiac people may enjoy a safe, nutritious, tasty and affordable diet. Three controversial issues have emerged: the high cost of a GF diet (GFD), the widespread availability and variety of GF products and the possible lower nutritional value of a GF diet.

GF foods are sold at prices considerably higher than conventional foods. In Australia, for example, a GF healthy (i.e., providing the right supply of nutrients and calories) food basket is significantly more expensive compared to a gluten-containing healthy food basket, and is therefore unaffordable for the low–average income family [2]. In Canada, GF products can cost more than twice as much as ‘regular’ wheat-based products, with added extra costs such as shipping for GF specialty items [3]. In Chile, it has been estimated that the excess monthly cost of a GFD amounts to €80.00 [4], while in Greece it amounts to €48–112 [5]. In the U.K., on average, GF products are 159% more expensive [6] than regular ones (€4.82 versus €1.25 per kg); it has been estimated [7] that —gross of the public subsidies that may be provided by the NHS—a U.K. celiac consumer pays on average an extra €11 each week to keep their pre-diagnosis utility level, corresponding to 29% of their food budget.

With regard to the nutritional quality of a GFD, there have been some flaws reported with respect to a conventional diet, such as a higher content of fat, saturated fat, sugar and salt [6,8], and a lower content of proteins, fiber and vitamins [4,9,10]. Moreover, higher concentrations of heavy metals in blood and urine, especially arsenic and mercury, have been found among people following a GFD compared to people not following a GFD [11]. Overall, a GFD has been associated with potential health risks, such as [12] a deficiency of micronutrients, hyperlipidaemia, hyperglycaemia and coronary artery disease.

The ease of access to GF products has dramatically increased over the last 10 years, at least in more developed countries, due to the rapidly expanding market for these products. A 2017 article of the Financial Times [13] reported that the global gluten-free retail market has grown from $1.7 billion in 2011 to $3.5 billion in 2016, and is forecast to grow to $4.7 billion in 2020, with the U.S., Italy and the U.K. leading this growth. While such growth positively affects the availability and variety of GF products, it nevertheless may be associated with a negative fact—namely that the market growth has been triggered by the growing number of non-celiac people choosing GF products on the assumption that avoiding gluten can have positive health effects and helps in losing weight [14,15]. While there is no evidence of a GFD as a healthier option for the general population [16], its risks instead have been widely investigated [8,17,18]. Moreover, the high consumers’ willingness to pay for GF products could induce manufacturers and retailers to charge extra prices on these products, with negative economic effects on celiac consumers and on the level of public expenditure aimed at their support.

This article studies the market of GF pasta in Italy. At the base of a Mediterranean diet, pasta is an important food in Italy, being part of Italian culture and its gastronomic heritage; its consumption satisfies not only the nutritional needs but also the hedonistic and conviviality needs linked to food. The aim of the study is to assess the offer of GF pasta in supermarkets with respect to its ability to meet the needs of celiac people in terms of variety, prices and safety. In particular, the study tries to verify whether celiac people have an easy access to GF pasta, pay an affordable price, may choose among different tastes and nutritional characteristics and are guaranteed reliability by GF certifications.

## 2. Materials and Methods

### 2.1. Survey Design

Data on GF pasta were collected in a sample of food stores, using a cross-sectional design and a stratified random sampling. The surveyed stores were representative of all retail formats (discount, hypermarkets and supermarkets, as classified by the EU [19]), and of all the 20 main retail groups operating in the Italian food market [20]. The study was performed in three Italian provinces (Livorno, Naples and Salerno) representative of the Italian food retail industrial structure, which is characterized by a high concentration in central in northern areas of the country (Livorno in Tuscany), and a lower concentration in southern Italy (Naples and Salerno in Campania) [21,22]. A sample of 212 stores was surveyed, accounting for around one in four of overall stores in the three provinces [23]. With respect to retail formats, the sample included discounts, 24.5%; hypermarkets, 12.3% and supermarkets 63.2%.

### 2.2. Data Collection

In each store, data were collected through structured direct observation. Direct observation provides the primary advantage of richer information on product characteristics, particularly for all attributes associated with claims reported on the label. Such product characteristics are usually missing data in a commercial database collected automatically through store scanners [24,25].

Among different types of pasta habitually sold in food stores, we collected data on the commercial category of dry pasta and on spaghetti-shaped pasta—the top-selling type of pasta sold in Italy—that is very versatile in cooking and is the base for many traditional dishes [26]. We used dry spaghetti-shaped pasta as a proxy of the whole pasta category. In the following text, the term pasta indicates dry and spaghetti-shaped pasta. To achieve good representativeness of the sample, items on promotion were excluded and the recognition of the items in the surveyed stores was repeated one month later.

For each selling unit, we collected information on a set of product characteristics. GF pasta items on store shelves (262 items in total) were identified by their European Article Number or EAN code. The following data were collected: price (reported as euros per kg); packaging size (three sizes: medium, 0.50 kg; small, 0.40 kg; very small, 0.25 kg); supplier and brand name; processing methods and label information. Label information included:

- type of grains (single-grain pasta or pasta of a blend of different grains), including corn, rice, millet, buckwheat, quinoa, sorghum and amaranth;
- fiber of GF pasta (whole-grain flour or flour enriched in fiber);
- country-of-origin labels of grains (Italy; European countries; non-European countries);
- organic certification;
- GF products in the NR, and thus entitled to be reimbursed by the Italian NHS;
- GF pasta with AIC certification.

### 2.3. Empirical Model

A hedonic price model (HPM) was estimated in order to identify the premium price associated with different attributes of individual selling units. In economic models for differentiated products, any good can be thought of as a bundle of various quality attributes that contribute to its overall value [27]. Consequently, the observed market price depends on the (implicit) price of each attribute, which is linked to the consumer utility derived from that specific attribute [28,29,30]. HPM has been extensively used to analyze differentiated markets [31,32,33,34,35,36,37]. It has been used by environmental economists [38,39,40], in labor economics [41] and in agricultural economics [25,42,43,44,45]. No previous study has studied implicit prices for attributes of gluten-free food.

HPMs rely on the assumption that the value of the bundle of attributes of a product is reflected in its market price. According to this assumption, the price is affected by all characteristics of the good (internal and external attributes). Based on this theoretical framework, the product price (**Price**) is related through a **P** function to a vector **k** of product attributes (**X**_k_), as follows:

(1) $$\mathrm{Price}=P(X_{1},X_{2},\ldots ,X_{k}),$$

where **P** can be different functional forms.

In our model specification, **Price** (dependent variable) was expressed in euros per kg and exploratory variables were dummy variables that took the value 0 or 1 in the absence or presence of each product characteristic. In HPMs, the hedonic price function (**P**) is not defined in advance and can take virtually any shape. On the basis of each specific case study and available data, the appropriate functional form of **P** was one of the outcomes of the empirical analysis, known in the literature as result of the first-stage estimation [30]. In our study, we estimated alternative functional forms suggested by the procedure of single-parameter Box–Cox transformation of the dependent variable (Price). The non-negativity of this variable was a pre-condition for statistical correctness of the Box–Cox transformation. Such a transformation was used because of the right-skewed distribution of the **Price** variable [46,47]. Among the set of forms suggested by the Box–Cox procedure, the cubic root functional form was preferred, assuring normalization of the dependent variable (and the error term) and the best possible fit of the model.

Finally, the following HPM was estimated:

(2) $${\mathrm{Price}}^{\lambda }{}_{j}^{}=\alpha_{0}+\sum_{a=1}^{2}\beta_{a}PS_{a,j}+\sum_{b=1}^{2}\beta_{b}BR_{b,j}+\sum_{c=1}^{3}\beta_{c}LB_{c,j}+\sum_{d=1}^{3}\beta_{d}PF_{d,j}+\sum_{e=1}^{8}\beta_{e}IN_{e,j}+\varepsilon_{j}^{},$$

where $\alpha_{o}$ is the intercept; $PS_{a}$ is the set of two attributes of pack size; $BR_{b}$ is the set of two attributes of brands; $LB_{c}$ is the set of three attributes of GF certification and claim; $PF_{d}$ is the set of three attributes of product features or production process characteristics; $IN_{e}$ is the set of eight attributes of main ingredients; $\varepsilon_{j}$ is the error term, independent and identically distributed according to a normal distribution N∼(0,1); **j** is the number of 262 items of GF spaghetti-shaped pasta and λ is the coefficient of Box–Cox transformation.

An ordinary least squares (OLS) method was applied to estimate the parameters of the linear regression model. Diagnostic tests assessing homoskedasticity (Breush–Pagan; Goldfeld–Quandt) and normality (Shapiro–Wilk, Jarque–Bera) were run.

## 3. Results

Table 1 reports descriptive statistics of the general data base. GF products are sold in 44.3% of retailers in the sample. They amount to 14% of the total pasta items, demonstrating that GF pasta is probably purchased by a public much larger than the 0.34% celiac people share of Italian population. As expected, the average price of GF pasta (€5.8) is about 2.5 times higher than the average price of the conventional product (€2.02). Price variability among GF pasta items is very high, with prices ranging from 2.18 to 8.36 per kg. Table 2 reports the frequencies of the quality attributes that characterize the GF pasta sample items.

The most popular GF pasta is made of mixed raw agricultural ingredients, with 51.5% of items made of corn and rice and 29.8% of corn, rice and one other ingredients (among the following: quinoa, buckwheat, amaranth, millet and sorghum). Only 8.4% of items use Italian raw agricultural ingredients, and the majority have a GF certification, offered either by the HM or by the AIC. Of the items, 35.9% are fiber-enriched and 16% are organic. Most of the products are sold in 400 g packs, and 18% are bronze drawn (where bronze drawn refers to a particular processing technique used for high quality dried pasta).

The variables used for estimating the price hedonic model, built from the elemental variables of the general dataset, are listed in Table 3, with the assigned code denominations.

The hedonic price model was estimated choosing as standard reference product (SRP) the GF items that are made of a mix of raw agricultural ingredients (namely corn and rice) and that do not possess any of the quality attributes codified in Table 3 and are used as independent variables. In Table 3 the characteristics of the SRP are also mentioned.

Starting from a model including all the variables in Table 3, the hedonic price function was estimated using a step-by-step procedure where non-significant variables were excluded in every successive estimation step. The final model is presented in Table 4. Of the investigated variables, 8 out of the 18 turned out to be significant, explaining 73% (adjusted R^2^ = 0.73) of the variance of the dependent variable. Three variables (production area of grains: Italy; one GF claim: presence in the NR; double GF claims: AIC certification and NR) exhibit a negative sign, while the other five (very small size; brand specialized in GF food; fiber-rich GF pasta; quinoa in multi-grain flour; single-grain flour of buckwheat) have a positive sign, indicating the existence of a premium price for the attributes that they represent.

## 4. Discussion

The results of statistical analysis allow for a quite general assessment of the capability of the Italian GF pasta market to meet the needs of celiac people, in particular with respect to availability, cost, variety, safety and market competitiveness.

GF pasta is sold only in 44% of the 212 stores of the sample (Table 1), which means a quite poor performance in terms of availability, considering also that pasta is a staple product, especially in the Mediterranean diet. The average price for GF pasta is €5.08 per kg, much higher than the average price of conventional pasta, which is €2.02 for stores offering GF pasta and €1.66 for the remaining stores. These findings are consistent with those of previous studies [4,48,49,50] that, all over the world, have reported the difficulty of access and the high economic burden of a GFD.

The study of product assortment helps to better understand the GF pasta market. GF pasta selling units amount to 14% of total pasta units (with and without gluten) of the whole sample and to 24% of units present in the 94 stores offering GF pasta. Since celiac people represent around 0.4% of the Italian population and people suffering from non-celiac gluten sensitivity (NCGS) range from 0.6 to 13% of the general population [51,52], the high share of GF items demonstrates that people without gluten related disorders (GRDs) also buy GF pasta. Such a result confirms the widely-reported phenomenon of many people avoiding gluten simply for trying new ways of eating that are perceived as healthier. People with no GRDs eat GF food because they believe (notwithstanding the lack of sound evidence) that it is healthier, useful for losing weight and helps to improve performance in sports [53,54]. The habit of avoiding gluten may entail harmful societal effects. First, many studies agree on the potential negative health effects of a GFD, due to the lower nutritional value [55] of GF foods (less protein, more glucose and saturated fats, less fiber) and the higher content in heavy metals [11]. Second, a GFD has a negative economic impact, because of the much higher prices of GF products, which is particularly burdensome for less wealthy people. Third, the wide consumption of GF products may damage celiac people in at least two ways: it can trigger excessive price rises, and it can trivialize celiac disease with the consequences of a weaker prevention of gluten contamination for GF products and of worsening the negative psychological effects of adherence to a strict GFD.

The many selling units of GF pasta mean a high assortment variety both in terms of brands and product characteristics. In our sample, GF products have a brand positioning similar to conventional pasta, with the lowest price for private label, an intermediate-high price for national brands and the highest prices for the other brands (Table 1), wherein we refer to national brands as those brands sold throughout the national territory and known by the general public and to private labels as the brands owned by retailers.

GF pasta items differ with respect to various characteristics (Table 2) which are either intrinsic (such as the kind of raw agricultural ingredient, a high fiber content or a particular processing system) or extrinsic (such as the presence of claims regarding the absence of gluten, the organic certification or the packaging size). The majority (85%) of selling units are made of a mix of grains and other agricultural ingredients, generally corn and rice (52%). Of the units, 36% are enriched with fiber, 16% have organic certification, 89% have AIC certification, 49% are sold in 0.4 kg packages and 85% are present in the NR of the IMH.

Items with different quality attributes exhibit different prices, which vary from a maximum of €7.9 per kg for pasta made of rice, quinoa and amaranth, to a minimum of €4.0 for the private label (PL) product.

The large variability of prices associated with quality attributes indicates that the market for GF pasta is highly differentiated. According to the economics of product differentiation [56,57], a differentiated market exhibits a non-competitive structure, with possible social welfare losses. These losses depend either on the possibility of market power (i.e., producers setting prices higher than marginal cost) or with a non-optimal size of production facilities, which does not allow for the exploitation of the economies of scale of the production process. Nevertheless, differentiation may also increase consumer welfare because of the larger product variety meeting consumers’ preferences and needs.

Product differentiation mirrors market segmentation, with some consumers willing to pay higher prices for certain product characteristics. Finding out which characteristics are most valued helps to understand consumers’ attitudes and to assess market performance.

The results of the estimation of the hedonic price function (Table 4) show that eight out of the 18 codified product characteristics explain 74% of price variability of GF pasta units in the sample. In Table 4, the fourth column (IP) reports the implicit price estimated for the associated variables, and the third column shows the estimate of the percentage change in the price of the SRP due to the presence of the product characteristic defined by the variable. For example, when the SRP is sold in 0.25 kg packages, its price increases by 51.1%.

Three variables were identified as able to decrease the price of the “standard product” (defined as the product which does not exhibit any of the investigated quality attributes): the claim of the Italian origin of the agricultural raw ingredient, the claim on the presence of the product in the NR together with the AIC certification and the sole claim of the presence in the NR. The negative value given to ‘made in Italy’, in contrast with what usually happens, may be due to the perception of a reduced GF specificity of the product that seems to be advertised as any other conventional pasta. The negative signs for the presence of the NR and the AIC certification mean instead that consumers of GF product need to be reassured about the reliability of the GF attribute; when such reliability is guaranteed by the IMH and the AIC, they do not need to pay an extra price (measured by the higher price of non-certified products) associated with the alleged cost borne by producers to guarantee and signal the GF character reliability. Another explanation could be that products with GF certification are mostly purchased by the very celiac people who try to buy less-expensive products while willing to be sure of their reliability.

Five variables were identified as able to receive a premium price. Small packages (0.25 kg) receive a premium price of 51.1%, seemingly because they are more convenient for single consumers and because the lower price per pack gives the false perception of saving money. Brands specialized in GF products also receive a high premium price (31.4%), probably as consumers recognize their products as of higher quality with respect to those offered by national brands and retailers that entered the market of GF products only recently. A high content in fiber is also well valued, signaling the consumers’ awareness of their nutritional needs. The willingness to pay higher prices for pasta containing quinoa (22.9% premium price), or made from a single ingredient such as buckwheat (24.5% premium price), indicates the interest in tastes and nutritional features different from those of the generally widely-consumed corn and rice pasta.

## 5. Conclusions

In Italy, celiac people are provided with state aid for covering the extra cost they incur to buy GF products. The well-being of celiac people depends on the availability, affordability, reliability and quality level—in terms of taste, variety and nutritional attributes—of GF products. Our study demonstrated that in the case of GF pasta, the availability is still limited, since the majority of surveyed retailers did not offer GF pasta. Affordability is also limited, due to high prices, while the reliability is high, thanks to the certifications provided by the IMH and the AIC. Product quality level is also high, due to the great variety of attributes and brands of the different selling units. Such a variety might entail a downside for people bounded to a GFD, as it may trigger price increases and trivialize the consumption of GF products that are increasingly chosen also by people not suffering from any form of gluten sensitivity. Our findings indicate that the recent surge in the GF market is associated with non-competitive market structures and might serve more to raise retailers’ profitability than to meet the needs of celiac people.

Our study encompassed a limited number of stores and locations. Interesting research developments could focus on price differences between different store sizes and locations, confronting, for example, urban and rural areas. A comparison across countries and the study of e-commerce [58,59] specificity for GF food markets could also be interesting objectives for further research.

## Figures and Tables

**Table 1:** Descriptive statistics of the sample. GF = gluten-free.

Stores	Stores		Items on Store Shelves			Price (€/kg)		
	n.	%	n.	%		Mean	SD ^a^	Min–Max
**Stores without GF pasta**	**118**	**55.7**	**818**		**43.2**	**1.66**	**0.63**	**0.64–5.38**
**Gluten-containing pasta**			**818**	**100**		**1.66**	**0.63**	**0.64–5.38**
National brand			532	65		1.87	0.61	0.64–5.38
Private label			222	27.1		1.25	0.41	0.65–2.38
Regional brand			64	7.8		1.43	0.6	0.75–3.80
**Stores with GF pasta**	**94**	**44.3**	**1074**		**56.8**	**2.77**	**1.85**	**0.60–7.98**
**Gluten-containing pasta**			**812**	**100**	**43.0**	**2.02**	**1.21**	**0.60–8.36**
National brand			610	75.1		1.9	0.81	0.60–6.90
Private label			156	19.2		1.59	0.98	0.75–5.08
Regional brand			46	5.7		5.11	1.9	0.82–7.98
**Gluten-free pasta**			**262**	**100**	**14.0**	**5.08**	**1.53**	**2.18–8.36**
National brand			144	55		5.02	1.51	2.85–7.96
Private label			50	19.1		3.99	1.15	2.18–7.56
Brand specialized in GF food			68	26		6	1.24	3.68–8.36
**Total stores**	**212**	**100**	**1892**		**100**			

Legend: ^a^ standard deviation (SD).

**Table 2:** GF selling units: product characteristics, prices and frequencies.

Description	Price (€/kg)		Frequencies	
	Mean	SD ^a^	n.	%
GF spaghetti-shape pasta on the store shelf	5.08	1.53	262	100
**Single-grain flour**				
Corn	4.86	1.27	14	5.3
Rice	5.81	0.69	12	4.6
Buckwheat	6.93	0.74	12	4.6
**Multi-grain flour**				
Corn and rice	4.6	1.69	135	51.5
Corn, rice and quinoa	5.21	0.66	48	18.3
Corn, rice and buckwheat	5.61	0.4	12	4.6
Corn, rice and millet	4.89	0.23	8	3.1
Rice, quinoa and amaranth	7.9	0.52	11	4.2
Corn, rice, quinoa and buckwheat	5.45	0.46	6	2.3
Corn, rice, buckwheat and sorghum	6.18	0.89	4	1.5
**Fiber-rich pasta** (whole-grain flour or added fiber)				
Fiber-rich products	5.85	1.01	94	35.9
Non fiber-rich products	4.65	1.6	168	64.1
**Dry pasta production**—wire-drawing technique				
Pasta drawn in bronze die-plates	5.26	0.63	46	17.6
Pasta drawn in Teflon die-plates	5.04	1.66	216	82.4
**Country-of-origin of grains**				
Italy	5.6	1.75	22	8.4
European Union or other countries	5.03	1.50	240	91.60
**Organic certification of European Union**				
Certified products (voluntary label)	6.21	1.05	42	16
Non-certified product	4.86	1.51	220	84
**Italian Association for Celiac Disease (AIC) certification**				
Certified products (voluntary label)	5.14	1.54	232	88.5
No certified products	4.58	1.37	30	11.5
**Product in the National Register (NR)** entitled to be reimbursed by the Italian National Health Service (NHS)				
Refundable products	4.97	1.45	223	85.1
Non-refundable products	5.69	1.84	39	14.9
**Brand**				
National brand	5.02	1.51	144	55
Private label	3.99	1.15	50	19
Brand specialized in GF food	6.00	1.24	68	26
**Pack sizes**				
Medium size (0.50 kg)	4.08	1.01	64	24.4
Small size (0.40 kg)	4.65	1.12	128	48.9
Very small size (0.25 kg)	6.77	1.20	70	26.7

Legend: ^a^ standard deviation (SD).

**Table 3:** Variable codification for the hedonic price model.

Variables		
Code	Description	Values
**Pack size**		
**SRP ^a^**	**Medium size (0.50 kg)**	
**PS1**	Small size (0.40 kg)	**1** if pack size of pasta is 0.40 kg; **0** otherwise
**PS2**	Very small size (0.25 kg)	**1** if pack size of pasta is 0.25 kg; **0** otherwise
**Brand**		
**SRP ^a^**	**National brand**	
**BR1**	Private label	**1** if brand of retailer; **0** otherwise
**BR2**	Brand specialized in GF food	**1** if specialized manufacturer; **0** otherwise
**Gluten free certification and claim**		
**SRP ^a^**	**No GF claim**	
**LB1**	Double GF claims: AIC and NR	**1** if pasta is in NR and has AIC certification; **0** otherwise
**LB2**	One GF claim: presence in the NR	**1** if pasta is in NR; **0** otherwise
**LB3**	One GF claim: AIC certification	**1** if pasta has AIC certification; **0** otherwise
**Product features or production process characteristics**		
**SRP ^a^**	**Non fiber-rich; non-organic; production area of grains: European Union (EU) and/or non-EU countries**	
**PF1**	Fiber-rich GF pasta	**1** if fiber-rich product; **0** otherwise
**PF2**	Organic GF pasta	**1** if organic; **0** otherwise
**PF3**	Production area of grains—Italy	**1** if Italy; **0** otherwise
**Main ingredients**		
**SRP ^a^**	**Two-grain flour: corn and rice**	
**IN1**	Single-grain flour: corn	**1** if corn as sole ingredient; **0** otherwise
**IN2**	Single-grain flour: rice	**1** if rice as sole ingredient; **0** otherwise
**IN3**	Single-grain flour: buckwheat	**1** if buckwheat as sole ingredient; **0** otherwise
**IN4**	Quinoa in multi-grain flour	**1** if quinoa mixed in a multi-grain flour; **0** otherwise
**IN5**	Amaranth, or millet, sorghum in flour mix	**1** if amaranth, millet, sorghum in flour mix; **0** otherwise
**IN6**	Buckwheat in multi-grain flour	**1** if buckwheat in a multi-grain flour; **0** otherwise
**IN7**	Rice in multi-grain flour (without corn)	**1** if rice in a multi-grain flour; **0** otherwise
**IN8**	Rice and corn in multi-grain flour	**1** if rice and corn in a multi-grain flour; **0** otherwise

Legend: ^a^ standard reference product (SRP).

**Table 4:** Estimated hedonic price model.

Variables		Model			Estimated	
					Implicit Price (IP)	
Code	Description	Coefficients		SE ^a^	%	IP (€/kg)
	Constant	1.426	***	0.019	-	4.08
**PS2**	Very small size (0.25 kg)	0.282	***	0.017	51.1	2.08
**BR2**	Brand specialized in GF food	0.185	***	0.018	31.4	1.28
**PF1**	Fiber-rich GF pasta	0.118	***	0.018	19.1	0.78
**PF3**	Production area of grains—Italy	−0.099	**	0.028	−13.8	−0.56
**LB1**	Double GF claims: AIC certification and NR	−0.089	***	0.020	−12.5	−0.51
**LB2**	One GF claim: presence in the NR	−0.092	*	0.039	−12.9	−0.53
**IN3**	Single-grain flour of buckwheat	0.148	**	0.050	24.5	1.00
**IN4**	Quinoa in multi-grain flour	0.139	***	0.018	22.9	0.93
	R^2^	0.737				
	Adjusted R^2^	0.729				
	F-test	88.824 ***				
	Observations (n.)	262				

Legend: significant at level * 0.05, ** 0.01, *** 0.001; ^a^ standard error (SE).
